# 

**Published:** 1878-12

**Authors:** 


					﻿THE BUFFALO
MEDICAL AND SURGICAL
JOURNAL.
VOL. XVIII.
DECEMBER, 1878.
NO. n.
				

